# Acute Decompensated Heart Failure and Cardiogenic Shock Following Propofol Infusion: A Report and Review of Pathophysiology

**DOI:** 10.7759/cureus.41815

**Published:** 2023-07-13

**Authors:** Abhinav Karan, Naji Maaliki, William M Kogler, Khadeeja Esmail

**Affiliations:** 1 Internal Medicine, University of Florida College of Medicine, Jacksonville, USA; 2 Cardiology, University of Florida College of Medicine, Jacksonville, USA

**Keywords:** acute cardiogenic pulmonary edema, acute decompensated heart failure, heart failure with reduced ejection fraction, ejection fraction, cardiogenic shock, propofol, heart failure

## Abstract

Propofol is a widely used general anesthetic agent with a generally familiar and predictable adverse effect profile. Severe left ventricular dysfunction to an ejection fraction of < 35% is a rare adverse effect of propofol, with a scarcity of data in the literature. In this case, we report a 36-year-old female at 36 weeks gestation with a prior remote history of peripartum cardiomyopathy, who, while receiving propofol for general anesthesia during a C-section, developed severe left ventricular dysfunction with an ejection fraction of 20-25%, flash pulmonary edema, and cardiogenic shock. She required initiation of inotropic support and, following weaning of propofol, gradually recovered her ejection fraction over the next 24 hours to 40-45% and to 50-55% at follow-up two weeks after discharge. This case highlights a unique adverse effect of propofol with scarce pre-existing literature and no guidelines on appropriate management. It is essential for clinicians to be familiar with this uncommon complication, particularly as propofol use continues to rise worldwide.

## Introduction

Propofol is a general anesthetic widely used for adults, including peripartum patients. While its multifaceted mechanism is believed to increase the GABA-mediated inhibitory tone within the central nervous system. While the adverse effects of hypotension and bradycardia are well-recognized in clinical practice, these are usually transient and rapidly improve without any need for intervention [1]. Left ventricular dysfunction has been seldom reported in clinical practice [2,3], with no guidelines on its appropriate management or pathophysiological mechanism. We report a previously healthy 36-year-old female at 36 weeks gestation who developed severe left ventricular systolic dysfunction with an ejection fraction of 20-25%, flash pulmonary edema, and cardiogenic shock after receiving propofol for general anesthesia for a cesarean section.

## Case presentation

A 36-year-old G5P4004 female at 36 weeks gestation presented for pre-delivery optimization before a scheduled cesarean section. She had a history of peripartum cardiomyopathy in her last cesarean delivery 6 years prior, with a reduced ejection fraction (EF) of 25-30%, which had recovered to 50-55% after goal-directed medical therapy with lisinopril, that had since been discontinued for the past 5 years. She was asymptomatic in the intervening years, had an excellent functional status with a Duke Activity Status Index of 58.2, and was pre-emptively admitted for pre-delivery risk stratification, given her prior history of peripartum cardiomyopathy and advanced maternal age. 

On presentation, she denied shortness of breath, orthopnea, bendopnea, paroxysmal nocturnal dyspnea, palpitations, or chest pain. She was euvolemic on physical examination with no evidence of pulmonary edema, jugular venous distension, or lower extremity edema. Her baseline NT-pro-BNP was 192pg/ml [reference range 0 - 125pg/ml]. A transthoracic echocardiogram revealed an ejection fraction of 50-55%, with no abnormalities. She was deemed low-risk from a cardiac standpoint and was scheduled for a C-section with epidural anesthesia.

While the patient was in her regular state of health before the C-section, immediately following the administration of epidural anesthesia with bupivacaine, she became acutely agitated and disruptive. At the time, delivery was considered urgent, and the decision was made to sedate and intubate the patient for an emergent C-section. She was given a bolus of 150 mg of propofol, followed by an infusion at 35 mcg/kg/min. However, 20 minutes following the initiation of propofol, the patient became hypotensive with an acute reduction in mean arterial pressure to 56 and hypoxic, requiring up-titration of FiO2 to 100% and initiation of an epinephrine infusion. An immediate bedside transthoracic echocardiogram (TTE) revealed an ejection fraction of 10-15% with global hypokinesis. She was transferred to the cardiac care unit after delivery for further management. Her exam revealed bilateral diffuse rales and an S3 gallop. A chest x-ray revealed diffuse pulmonary edema. A TTE demonstrated severe left ventricular systolic dysfunction with EF of 20-25% and global hypokinesis (Video 1). As the patient's propofol infusion was weaned off, her MAP improved rapidly, allowing the rapid discontinuation of her epinephrine infusion.

**Video 1:** Apical Four Chamber View of transthoracic echocardiogram revealing severely reduced ejection fraction of 20-25% following administration of propofol.

The patient rapidly improved and was extubated to bilevel positive pressure ventilation shortly after. She continued a short course of bolus diuresis leading to a resolution of pulmonary edema and improvement in EF to 40-45% within 24 hours of admission to the cardiac care unit. She was discharged successfully on day 2 following admission on losartan 25 mg once daily and metoprolol succinate 50 mg once daily. On outpatient follow-up, two weeks after discharge, her EF on TTE was improved to 50-55%, and she was well-compensated, with no further complaints. 

The differential diagnoses of stress-induced cardiomyopathy and recurrence of peripartum cardiomyopathy were considered; however, the diagnosis of propofol-induced LV dysfunction was made based on the rapid acuity of LV systolic dysfunction with the initiation of intra-operative propofol, alongside the rapid improvement of LV systolic function with cessation of propofol.

## Discussion

Our case represents a case of acute decompensated heart failure with reduced ejection fraction in a young female undergoing Cesarean section shortly after the initiation of propofol. The acute onset of LV systolic dysfunction and the rapid recovery of her ejection fraction after discontinuation supported a diagnosis of propofol-induced LV dysfunction. While recurrence of peripartum cardiomyopathy and stress-induced cardiomyopathy remained on the differential diagnoses, they were considered less likely due to a lack of recurrent postpartum cardiomyopathy in interim pregnancies and the onset of LV systolic dysfunction acutely following propofol infusion. Furthermore, while the meantime for recurrence of peripartum cardiomyopathy is within 2.2 years, our patient presents 6 years following her last occurrence of peripartum cardiomyopathy [4]. Our case aims to highlight the scenario of propofol-induced LV systolic dysfunction and cardiogenic shock. Propofol is an intravenous anesthetic agent commonly used in clinical practice to maintain general anesthesia. It is popular owing to its rapid, dose-dependent onset of action, large volume of distribution, and rapid recovery from clinical effects [1].

It is prudent for clinicians to be aware of adverse outcomes from propofol use. Propofol can result in hypotension and bradycardia due to a sympatholytic effect at clinical doses, which typically is transient and requires vasoactive support in less than 0.2% of all cases [1]. One study estimates an incidence of hypotension (systolic blood pressure <90 mmHg) of 15.7%, bradycardia at 4.8%, and both at 1.3% [1]. It induces a transient but rapid decrease (15%) in mean arterial pressure (MAP) exclusively related to a decrease in cardiac index (CI) without reduction in indexed systemic vascular resistances (SVRI) [2]. 

In higher doses, propofol has been shown to directly impair myocardial contractility [2]. Propofol has been shown to cause a concentration-dependent decrease in maximal developed tension in myocardial musculature, statistically significant at supratherapeutic concentrations exceeding the clinical range at which propofol is typically used [3]. While hypotension exhibited by our patient is not uncommon, sustained hypotension with the requirement for initiating vasoactive support is exceedingly uncommon. Furthermore, although propofol was used in therapeutic concentrations, we can postulate, given the clinical presentation, that our patient's severe LV dysfunction with subsequent flash pulmonary edema resulted from a sudden reduction in myocardial contractility and the sympatholytic effect of propofol. The rapidity of the effect of propofol to cause hypotension through the relaxation of vascular smooth muscle cells has previously been established, but the mechanism behind a sustained hypotensive response is much less clear and requires further investigations into the pharmacokinetic properties of propofol [5]. Pharmacodynamic studies on propofol have revealed that it acts at a cellular level as a calcium antagonist by reducing vascular tone, depressing myocardial contractility, and inhibiting compensatory tachycardia [4,5]. A link between propofol infusion and our patient's cardiac dysfunction was made as our patient's EF, mean arterial pressure, and diffuse pulmonary edema improved rapidly following the cessation of propofol, and she remains well-compensated on outpatient follow-up. 

LV dysfunction from propofol is an extremely important consideration in peri-operative management. Given the ability of propofol to depress left ventricular systolic dysfunction, it is possible that it may result in clinical presentations of peripartum cardiomyopathy or as an agent that can exacerbate stress cardiomyopathy. It is reasonable, particularly those patients with a prior cardiac history of LV dysfunction, to use propofol judiciously to avoid this notable adverse outcome on patient care due to the paucity of data available on propofol's effect on myocardial contractility, further prospective studies and necessary to elaborate clinical recommendations from these findings.

## Conclusions

Left ventricular dysfunction from propofol use is an uncommon complication requiring increased awareness in peri-operative management. While propofol is a widely used anesthetic agent with a generally predictable adverse effect profile, we propose that in patients with pre-existing left ventricular dysfunction, propofol be used judiciously with a standardized management protocol should LV dysfunction arise. Due to the paucity of existing literature on propofol's effect on myocardial contractility, further randomized controlled trials are necessary to elucidate more data on this uncommon effect.
